# An In Silico Modelling Approach to Predict Hemodynamic Outcomes in Diabetic and Hypertensive Kidney Disease

**DOI:** 10.1007/s10439-024-03573-2

**Published:** 2024-07-05

**Authors:** Ning Wang, Ivan Benemerito, Steven P Sourbron, Alberto Marzo

**Affiliations:** 1 INSIGNEO Institute for In Silico Medicine, The University of Sheffield, Sheffield, UK; 2 Department of Mechanical Engineering, The University of Sheffield, Sheffield, UK; 3 School of Medicine and Population Health, The University of Sheffield, Sheffield, UK; 4 The University of Sheffield, Room E09, The Pam Liversidge Building, Mappin Street, Sheffield, S13JD UK

**Keywords:** Chronic kidney disease, Hypertension, Diabetes mellitus, 1D modelling, Renal circulation modelling, Biomarkers

## Abstract

Early diagnosis of kidney disease remains an unmet clinical challenge, preventing timely and effective intervention. Diabetes and hypertension are two main causes of kidney disease, can often appear together, and can only be distinguished by invasive biopsy. In this study, we developed a modelling approach to simulate blood velocity, volumetric flow rate, and pressure wave propagation in arterial networks of ageing, diabetic, and hypertensive virtual populations. The model was validated by comparing our predictions for pressure, volumetric flow rate and waveform-derived indexes with in vivo data on ageing populations from the literature. The model simulated the effects of kidney disease, and was calibrated to align quantitatively with in vivo data on diabetic and hypertensive nephropathy from the literature. Our study identified some potential biomarkers extracted from renal blood flow rate and flow pulsatility. For typical patient age groups, resistive index values were 0.69 (SD 0.05) and 0.74 (SD 0.02) in the early and severe stages of diabetic nephropathy, respectively. Similar trends were observed in the same stages of hypertensive nephropathy, with a range from 0.65 (SD 0.07) to 0.73 (SD 0.05), respectively. Mean renal blood flow rate through a single diseased kidney ranged from 329 (SD 40, early) to 317 (SD 38, severe) ml/min in diabetic nephropathy and 443 (SD 54, early) to 388 (SD 47, severe) ml/min in hypertensive nephropathy, showing potential as a biomarker for early diagnosis of kidney disease. This modelling approach demonstrated its potential application in informing biomarker identification and facilitating the setup of clinical trials.

## Introduction

Chronic kidney disease (CKD) is a debilitating condition that affects approximately 800 million individuals worldwide. Its estimated annual medical costs vary across disease stages, ranging from approximately $1700 (stage 2), to $3500 (stage 3), and $12,700 (stage 4) per patient [1]. CKD is more frequently observed in older age groups, with a prevalence of 39.4% among individuals over 60 years old, compared to 12.6% and 8.5% among those aged 40–59 and 20–39 years, respectively [2]. Unfortunately, most CKD patients do not experience symptoms until the disease progresses to the later stages when the condition is irreversible [1]. Furthermore, CKD diagnosis is often confounded by different cause-effect mechanisms and co-morbidities, especially diabetes (affecting 40% of CKD patients) and hypertension (affecting 35.8% of stage 1 CKD patients, 84.1% of stage 4 and 5 CKD patients) [3, 4]. Hypertensive nephropathy (HN) is a kidney disease linked to prolonged high blood pressure, whereas diabetic nephropathy (DN) is a severe complication of diabetes marked by kidney damage due to sustained high blood sugar levels, both leading to similar CKD symptoms. Diabetes and hypertension are common comorbidities, but the only way to distinguish between HN and DN is through invasive kidney biopsies. In diabetic kidney disease, characteristic Kimmelstiel-Wilson lesions manifest in the glomerular capillary loops [5], whereas this feature is absent in hypertensive kidney disease [6].

Kidney biopsies are rarely performed in the earlier disease stages where there is most scope for renoprotective intervention. There exists therefore a critical unmet need for more accessible and non-invasive diagnostics to distinguish between these two main causes of CKD. Since diabetes and hypertension both have strong vascular involvement, one possible route is through hemodynamic imaging biomarkers, either from ultrasound (US) or magnetic resonance imaging (MRI). The US biomarker resistive index (RI) is readily available in clinical practice and is affected by kidney disease [7–10]. In hypertension, RI increases from 0.65 to 0.73 from stage 2 to stage 3 [11], and an elevated RI is related to renal organ damage [7]. Patients at stage 2 of HN, who were undergoing antihypertensive therapy, had RI in the normal range [8]. RI also increases in DN, reflecting progressive renal damage [9], with RI increasing from 0.66 at stage 1 to 0.85 at stage 5 [10]. An example of a hemodynamic biomarker measurable in MRI is the renal blood volumetric flow rate (RBF, in mL/min, measured by phase-contrast MRI) [12–14]. RBF distinguishes between healthy individuals and those with DN but there is no data on the correlation with estimated glomerular filtration rate (eGFR) in CKD [15, 16]. While biomarkers such as RBF and RI may have some use in distinguishing between HN and DN, it is difficult to put forward precise hypotheses without a clearer mechanistic understanding of the relationship between these different etiologies and their effect on the biomarkers.

In the last few decades, in silico medicine has established itself as a valuable tool for representing intricate, non-observable physiological and pathological mechanisms in the human body [17–19]. Reduced-order modelling approaches have gained significant popularity for their capacity to navigate the trade-off between computational efficiency and physiological accuracy. 1D modelling approaches can reliably describe the physics of pulse wave propagation within the cardiovascular system [20, 21]. These models can be naturally aligned with well-established clinical procedures by including the modelling of the whole systemic circulation [22], the effects of age on pulse waves [23], the cerebral circulation [24], and the pulmonary circulation [25]. Through the use of these models, and by simulating the effects of pathology on pressure waveforms, several authors have identified potential biomarkers for the diagnosis of many diseases, such as pulmonary hypertension [25], cerebral vasospasm [26], coronary artery disease [27], and aortic aneurysms [28].

Existing in silico investigations on kidney disease are focused on 3D patient-specific modelling of blood flow through specific regions of interest, such as the main renal artery [29, 30], as the larger lumen area of this vessel allows for easier image acquisition, segmentation, and geometry reconstruction. This can be useful for diagnosing nephropathy caused by renal artery stenosis, but offers limited insights into the representation and understanding of other essential comorbidities such as DN and HN, which can affect systemic circulation as well as the smaller renal vessels and microvasculature. Haemodynamic characteristics of intrarenal arteries undergo significant changes in kidney disease, and these changes, such as vessel stenosis and RI measured in the smaller segmental renal arteries, have been proposed in the diagnosis of kidney disease [31, 32]. A more comprehensive model representation of the more peripheral, smaller vessels within the kidneys, and the systemic effects of diabetes and hypertension would allow a more precise characterisation of CKD pathophysiology and offer an opportunity for validation and alignment with the published clinical data. This remains largely unexplored.

This study aimed to develop, validate, and calibrate a modelling approach that extended an existing 1D whole circulation model to include a more comprehensive renal circulation component, including the intrarenal vascular networks, where validation and calibration were conducted utilising in vivo data sourced from the literature. The model incorporates an ageing and a DN and HN model, across several stages of disease progression. The model also allowed the mechanistic representation of these conditions in virtual populations, for the representation of physiological variability and the identification of clinically-aligned, effective biomarkers that could be used to distinguish between HN and DN from an early stage.

## Materials and Methods

**Fig. 1:**
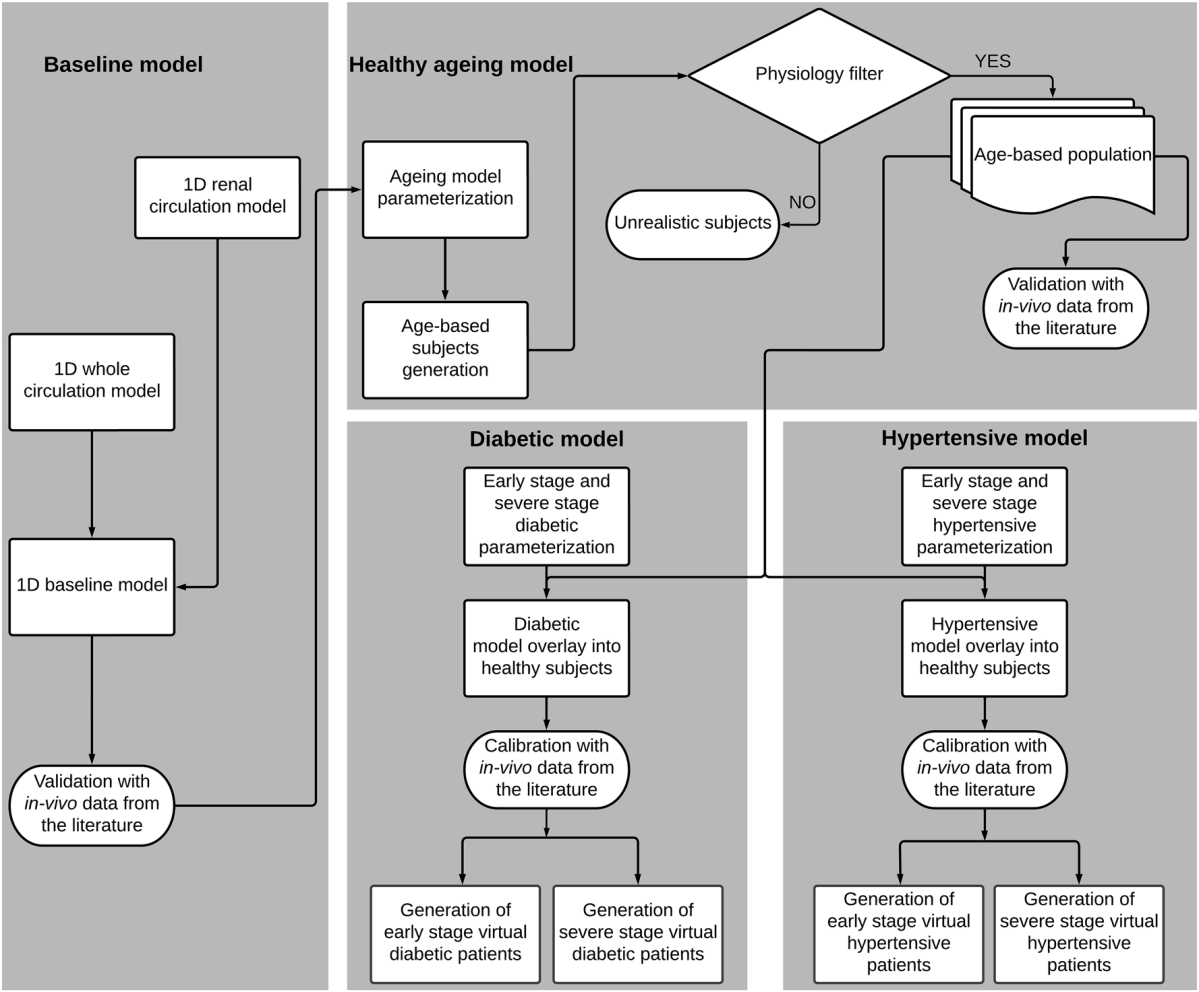
Workflow chart of the study methodology. Validation through comparison with in vivo data was performed for the baseline and healthy ageing model, whereas calibration was performed through comparison with in vivo data for the disease models

The workflow in Fig. 1 shows the different stages of development and parameterisation of the model, which is further described in the paragraphs below.

### 1D Whole-Circulation Baseline model

The network utilised in this study was extended starting from an existing, validated anatomical 1D model [22, 33]. The newly developed model, comprising a total of 113 blood vessels, one inlet (ascending aorta) and 49 outlets, includes additional branching of the abdominal aortic vessel into the left and right renal networks. A total of 38 renal arteries from the main renal arteries to the smaller interlobar arteries were added on each side of the descending thoracic aorta [34]. Fiugre 2 shows the anatomy and extent of the resulting circulation model. A comprehensive and detailed description of the model and its parametrization is available as supplementary materials.

**Fig. 2:**
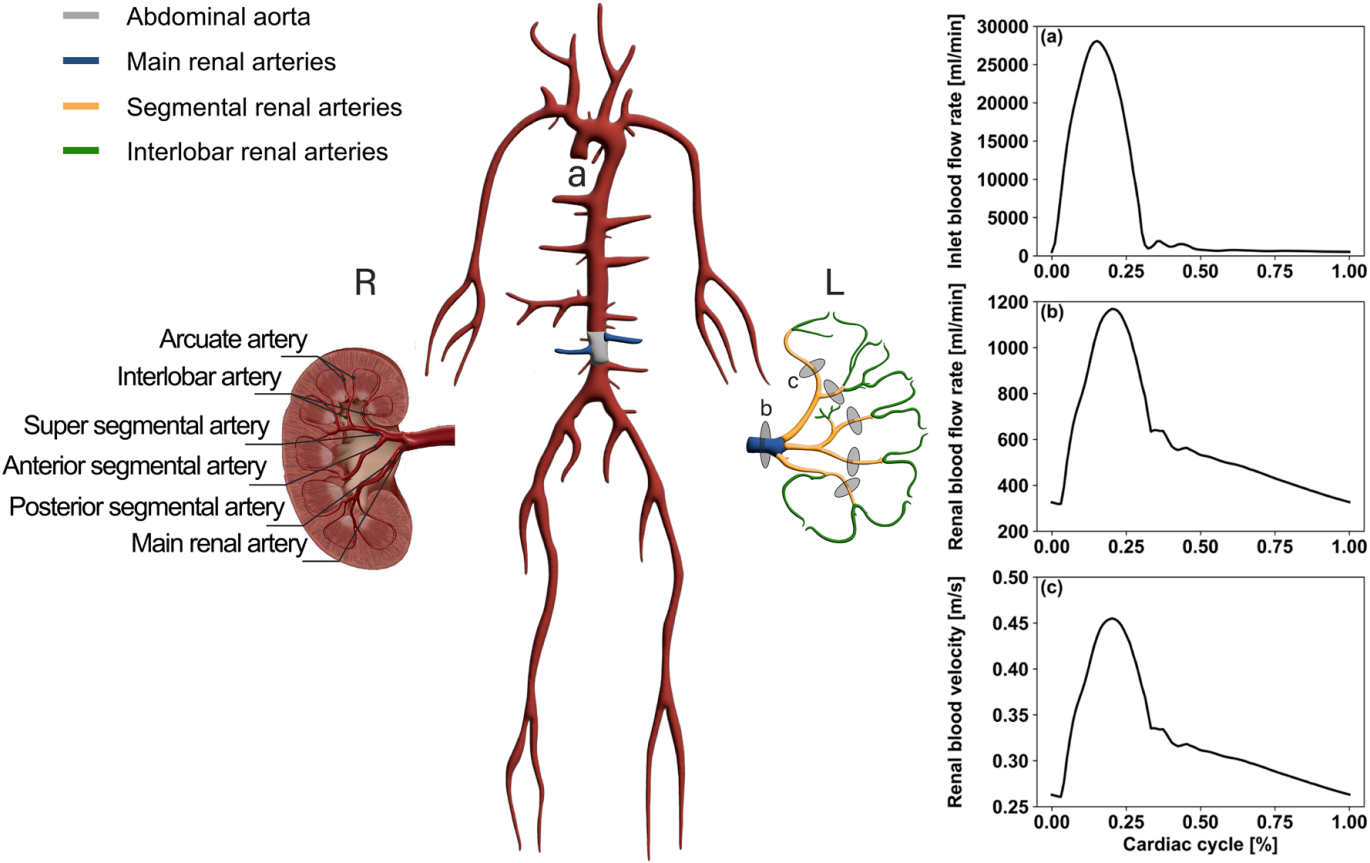
Illustration of openBF whole-circulation network (centre) with the two renal networks (left and right). Measurement locations of the mean RBF rate were shown by a grey circular plane (I) at the main renal artery, and measurement locations for blood velocity waveforms and RI values were shown by grey circular planes (II) at the five segmental renal arteries in the enlarged representation of the left (L) renal network. Model flow rate waveforms for a typical individual were shown on the right hand side, **a** blood volumetric flow rate in ascending aorta imposed as inlet boundary condition, **b** renal blood volumetric flow rate predicted at location (I), **c** renal blood velocity at location (II)

The model parameters included vessel geometry (lumen radius, length, and thickness), and material properties (Young’s modulus, Poisson’s ratio), where Young’s modulus represented blood vessel’s elasticity. Volumetric flow rates, derived from cardiac output (CO) data reported in the literature, were applied in the ascending aorta as an inlet boundary condition. Peripheral vascular resistance (PVR) and compliance (PVC), here representing viscous resistance to flow and compliance in the peripheral vascular bed, were described using lumped-parameter R-C-R (vascular resistance and compliance) models fully coupled with the 1D model at its outlets. In this study, the arcuate, interlobular arterioles, and glomeruli in the renal circulation were described using lumped-parameter R-C-R models. Furthermore, our model utilised generic parameters from the literature, aiming for gender neutrality. Each geometric and material parameter was assigned a single value that fell within the range observed for both males and females.

The Navier-Stokes-based open-source software, openBF [35], was employed to compute blood pressure, volumetric flow rate and pressure waveforms for a Newtonian fluid in each vessel throughout a complete cardiac cycle. Results were derived from the solution of the discretized form of the continuity (1), momentum (2) and constitutive (3) equations reported below, using the finite volume method [33]:

1 $$\frac{{\partial {\text{A}}}}{{\partial {\text{t}}}} + \frac{{\partial {\text{Q}}}}{{\partial {\text{z}}}} = 0$$

2 $$\frac{{\partial {\text{Q}}}}{{\partial {\text{t}}}} + \frac{\partial }{{\partial {\text{z}}}}\left( {{\upalpha }\frac{{{\text{Q}}^{2} }}{{\text{A}}}} \right) + \frac{{\text{A}}}{{\uprho }}\frac{{\partial {\text{P}}}}{{\partial {\text{z}}}} = - 2{\upmu }\left( {{\upgamma }_{{\text{v}}} + 2} \right)\frac{{\text{Q}}}{{\text{A}}}$$

3 $${\text{P}}\left( {\text{A}} \right) = {\text{P}}_{{{\text{ext}}}} + \upbeta \left( {\sqrt {\frac{{\text{A}}}{{{\text{A}}_{{0}} }}} - 1} \right), \upbeta = \sqrt {\frac{\pi }{{{\text{A}}_{{0}} }}} \frac{{{\text{Eh}}_{0} }}{{1 - \nu^{2} }}$$

where A is the vessel cross-sectional area, Q is the volumetric flow rate, $(\text{P}(\text{A}) -\text{ Pext})$ is the transmural pressure, ${\text{A}}_{0}$ is the reference cross-sectional area, ${\text{h}}_{0}$ is the reference wall thickness, $\text{E}$ is the vessel Young’s modulus, $\upnu$ is the Poisson’s ratio, $\uprho$ is blood density, $\upmu$ is blood viscosity, ${\upgamma }_{\text{v}}$ is a parameter that regulates the shape of the velocity profile for the calculation of viscous losses, and α is the Coriolis coefficient.

Velocity values across the cardiac cycle at each location, were derived from volumetric flow rates divided by the vessel cross-sectional area at the same locations. RI values were computed from (4) at five locations in the left and right renal networks, at segmental artery level, as shown in Fig. 2. These were reported as a mean representative value for all these locations. Mean RBF rates were calculated as arithmetic average of volumetric flow rate across a cardiac cycle.

4 $${\text{RI}} = \frac{{{\text{V}}_{{{\text{PSV}}}} - {\text{V}}_{{{\text{EDV}}}} }}{{{\text{V}}_{{{\text{PSV}}}} }}$$

where ${\text{V}}_{\text{PSV}}$ is peak systolic blood velocity, ${\text{V}}_{\text{EDV}}$ is end diastolic blood velocity.

### Ageing Model

The ageing process is known to influence various mechanical, physiological, and haemodynamic parameters such as length, lumen radius, Young’s modulus, wall thickness, PVR, PVC, and CO. The non-dimensional values of these properties, together with their variability range, were informed from data in the literature, assuming a Gaussian distribution within each age group, as presented in Table 1. In this study, we assumed that the parameters of each blood vessel within each circulation model were mutually independent. These parameters were randomly and independently assigned by sampling each value for each blood vessel from its respective Gaussian distribution. Using these data and their distributions within each age group, we parameterised our baseline model to represent the ageing process in an initial virtual population of 12,000 individuals (2,000 individuals per age group) ranging from 20 to 79 years old (yo). The actual dimensional values for age group 20-29 yo model parameters, can be found in the supplementary material, and were used to nondimensionalize all data within all age groups in Table 1. This resulted in the non-dimensional values, reported in Table 1, for CO (inlet boundary condition), and vessel mechanical properties for all vessels. The corresponding age-based healthy subjects were generated by multiplying the dimensional model parameters by the age-specific, non-dimensional scaling values. Some model parameterisations were filtered out (physiology filter, Fig. 1) if their solutions led to unphysiological values of systolic blood pressure (SBP) or diastolic blood pressure (DBP) (for details, see 2.6). Typical values for brachial pressure reported in the literature and in Table 1 were used to remove unrealistic predictions in the physiological filter.

**Table 1:** Distribution of normalised parameters for the ageing model

Parameters	Age groups [year old]					
	20-29 n = 759 Mean (SD)	30-39 n = 822 Mean (SD)	40-49 n = 701 Mean (SD)	50-59 n = 711 Mean (SD)	60-69 n = 655 Mean (SD)	70-79 n = 605 Mean (SD)
Cardiac output	1.00 (0.22)	1.00 (0.22)	0.91 (0.22)	0.87(0.21)	0.80 (0.20)	0.75 (0.18)
Length	1.00 (0.13)	1.08 (0.14)	1.16 (0.16)	1.22 (0.17)	1.32 (0.18)	1.40 (0.19)
radius	1.00 (0.03)	1.03 (0.06)	1.04 (0.07)	1.07 (0.07)	1.15 (0.10)	1.18 (0.11)
Young’s modulus	1.00 (0.13)	1.10 (0.18)	1.23 (0.16)	1.32 (0.25)	1.60 (0.35)	2.00 (0.45)
Thickness	1.00 (0.18)	1.10 (0.20)	1.12 (0.22)	1.25 (0.22)	1.51 (0.26)	1.75 (0.28)
PVR	1.00 (0.20)	1.06 (0.24)	1.15 (0.25)	1.25 (0.27)	1.23 (0.28)	1.46 (0.29)
PVC	1.00 (0.25)	0.89 (0.24)	0.77 (0.21)	0.65 (0.18)	0.58 (0.15)	0.50 (0.11)
Brachial pressure						
SBP (mmHg)	120 (11)	119 (11)	121 (11)	124 (11)	126 (10)	127 (10)
DBP (mmHg)	74 (8)	75 (8)	76 (7)	77 (7)	76 (7)	74 (7)

Distribution of scaling parameters for CO are from [23], length [23], radius [23], Young’s modulus [23, 36], thickness [23, 37], PVR and PVC [23] across age groups ranging from 20–29 to 70–79 years old. Number of virtual patients within each age group after the filtering process is reported as *n*. Typical distributions of brachial pressure are from [38]

### Kidney Disease Progression

In openBF we cannot directly simulate eGFR and urinary albumin-to-creatinine ratio (ACR), which are normally used to measure progression of kidney disease. Instead, disease stages were simulated by matching RI values to in vivo data from the literature linked to these stages. These values correspond with mild to moderate renal impairment in DN and HN patients. RI data (mean ± SD) used for establishing these four models include: mild renal function impairment (RI: 0.69 ± 0.05 [10]) for DN patients, moderate renal function impairment (RI: 0.74 ± 0.02 [10]) for DN patients, mild renal function impairment (RI: 0.65 ± 0.07 [8]) for HN, and moderate renal function impairment (RI: 0.73 ± 0.05 [11]) for HN. Parameterizations are shown in Tables 2 and 3**.** In the remainder of the text, we refer to these groups as: Early.D (early diabetes), Severe.D (severe diabetes), Early.H (early hypertension), and Severe.H (severe hypertension), respectively.

**Table 2:** Parameter distribution presented as mean and standard deviation (SD) for early and severe diabetic models

Parameters	Healthy	Early.D		Severe.D	
	Model data^a^ Mean (SD)	Normalised data Mean (SD)	Calibrated model	Normalised data Mean (SD)	Calibrated model
Viscosity (mPa·s)	4.00	~1.07 (0.26)	1.10	~1.23 (0.30)	1.20
Cardiac output (L/min)	5.74	0.85 (0.10)	0.85	0.72 (0.05)	0.70
Lumen radius (mm)					
Abdominal aorta	7.49	0.93 (0.02)	0.95	0.91 (0.02)	0.90
Renal artery	2.71	n/a	0.95	n/a	0.90
Carotid artery	3.17	0.93 (0.02)	0.95	n/a	0.90
Radial artery	1.38	0.96 (0.02)	0.95	0.96 (0.02)	0.90
Other arteries	0.68–12.95	n/a	0.95	n/a	0.90
Young’s modulus (kPa)					
Ascending aorta	400	1.15 (n/a)	1.10	1.29 (n/a)	1.30
Renal artery	400	n/a	1.10	n/a	1.30
Radial artery	400	1.06 (0.30)	1.10	n/a	1.30
Brachial artery	400	1.10 (0.28)	1.10	n/a	1.30
Carotid artery	400	1.18 (0.64)	1.10	n/a	1.30
Other arteries	400–500	n/a	1.10	n/a	1.30
Wall thickness (mm)					
Descending aorta	2.12	1.21 (0.23)	1.15	n/a	1.28
Renal artery	0.54	n/a	1.15	n/a	1.28
Carotid artery	0.70	1.05 (0.2)	1.15	1.40 (0.03)	1.28
Other arteries	0.34–1.79	n/a	1.15	n/a	1.28
Vascular bed					
PVR (10^10^·Pa·s/m^3^)	0.11–17.20	1.12 (0.26)	1.20	1.40 (0.33)	1.40
PVC (10^−10^·m^3^/Pa)	0.02–2.60	0.82 (0.25)	0.82	0.80 (0.26)	0.80

For early stage diabetes: distribution of normalised blood viscosity are from [39, 40]; Distribution of normalised CO [41]; Distribution of normalised lumen radius in abdominal aorta [42], carotid artery [42], and radial artery [42]; Distribution of normalised Young’s modulus in ascending aorta [43], radial artery [44, 45], brachial artery [44, 45], and carotid artery [46, 47]; Distribution of normalised wall thickness in descending aorta [48] and carotid artery [49]; Distribution of PVR [50–52] and PVC [53]. For severe stage diabetes: distribution of normalised blood viscosity [39, 40, 54]; Distribution of normalised CO [55]; Distribution of normalised lumen radius in abdominal aorta [42] and radial arteries [42]; Distribution of normalised Young’s modulus in ascending aorta [43]; Distribution of normalised wall thickness in carotid artery [42]; Distribution of normalised PVR [53] and PVC [53]

^a^Detailed values for those parameters reported as a range in the healthy model data are provided in the supplementary material

**Table 3:** Parameter distribution presented as mean and standard deviation (SD) for early and severe hypertensive models

Parameters	Healthy	Early.H		Severe.H	
	Model data^a^ Mean (SD)	Normalised data Mean (SD)	Calibrated model	Normalised data Mean (SD)	Calibrated model
Viscosity (mPa·s)	4.00	1.02 (0.09)	1.00	1.06 (0.12)	1.00
Cardiac output (L/min)	5.74	~1.15 (n/a)	1.15	n/a	1.00
Lumen radius (mm)					
Ascending aorta	15.95	0.96 (0.02)	0.95	0.92 (0.02)	0.93
Renal artery	2.71	1.00 (0.13)	0.98	n/a	0.95
Carotid artery	3.17	0.99 (n/a)	0.98	n/a	0.95
Other arteries	0.68–12.95	n/a	0.98	n/a	0.95
Young’s modulus (kPa)					
Ascending aorta	400	n/a	1.20	n/a	1.40
Renal artery	400	n/a	1.20	n/a	1.40
Carotid artery	400	1.21 (0.54)	1.20	1.51 (0.98)	1.40
Other arteries	400-500	n/a	1.20	n/a	1.40
Wall thickness (mm)					
Descending aorta	2.12	1.00 (0.10)	1.00	n/a	1.10
Renal artery	0.54	n/a	1.00	n/a	1.05
Carotid artery	0.70	1.02 (0.22)	1.00	n/a	1.05
Other arteries	0.34–1.79	n/a	1.00	n/a	1.05
Vascular bed					
PVR (10^10^·Pa·s/m^3^)	0.11–17.20	1.17 (0.27)	1.15	1.33 (0.32)	1.35
PVC (10^-10^·m^3^/Pa)	0.02–2.60	0.86 (0.25)	0.86	0.67 (0.02)	0.70

For early stage hypertension: distribution of normalised blood viscosity are from [56]; Distribution of normalised CO [57]; Distribution of normalised lumen radius in ascending aorta [58–60], renal arteries [61], and carotid artery [62]; Distribution of normalised Young’s modulus in carotid artery [46]; Distribution of normalised wall thickness in descending aorta [63] and carotid artery [62]; Distribution of normalised PVR [64] and PVC [65]. For severe stage hypertension: Distribution of normalised blood viscosity [66]; Distribution of normalised lumen radius in ascending aorta [59, 60]; Distribution of normalised Young’s modulus in carotid artery [67]; Distribution of normalised PVR [68] and PVC [65, 68]

^a^Detailed values for those parameters reported as a range in the healthy model data are provided in the supplementary material

### Diabetic Patient Model

Diabetes is known to influence the geometric and mechanical properties of blood vessels, such as their Young’s modulus, wall thickness, proximally and peripherally, and therefore PVR, and PVC. Additionally, high blood sugar (hyperglycemia) can increase blood viscosity due to the decrease of red blood cell fluidity and deformability, which in turn leads to a decrease in CO. As diabetes advances, these properties gradually change, and extensive quantitative data supporting these changes are documented in Table 2.

In order to link vascular properties to different stages of progression of diabetes, we conducted an analysis of the mechanical properties of blood vessels between healthy individuals and diabetic patients using data available in the literature (references in table caption). We categorised mild or moderate renal function impairment based on either the eGFR or the urinary ACR, as determined in each respective study. We did not consider the influence of age on disease progression as this information was often not available. Following this stratification, we proceeded to extract the data on vascular mechanical properties for differing degrees of renal dysfunction. In instances where specific blood vessels’ data were not available in the literature for diabetic patients, but those vessels were present in our model, we extrapolated their behaviour based on trends observed in other blood vessels, for which data was available. Additionally, data used in diabetic patient models at various disease stages were normalised using data available for healthy individuals.

Subsequently, model parameters were calibrated within their pathophysiological range, to achieve a better alignment with in vivo data on RI. This calibration process was informed by a global Sobol’s sensitivity analysis where the effects of variability of all model parameters (length, lumen radius, Young’s modulus, wall thickness, PVR and PVC) and its effect on RI was studied. The result of the sensitivity analysis showed that the lumen radius, PVR, and PVC of the renal arteries exhibited the highest sensitivity towards renal RI. Only these parameters were changed manually in the calibration process, while other less sensitive parameters were aligned with reliable pathological changes, until good alignment was achieved with the in vivo data. Specifically, we aimed to minimise the percentage difference in mean RI values between in vivo and virtual DN populations to below 5% at both early stages (in vivo: 0.69 [10] vs. openBF: 0.70) and severe stages (in vivo: 0.74 [10] vs. openBF: 0.76).

### Hypertensive Patient Model

Hypertension, through different mechanisms, has a similar impact on the geometric and mechanical characteristics of blood vessels as diabetes, although the extent and variability of these changes are different. During the earlier stages of hypertension, blood vessels activate short-term compensatory mechanisms such as vasodilation and adjustments in blood vessel compliance to counteract the increased pressure. This adaptive response aims to maintain normal blood flow and thus control blood pressure within the normal range. Over the longer term, arteries undergo remodelling in response to chronic pressure overload. This remodelling involves changes in the size, structure, and composition of the vessel walls. Over time, this can lead to concentric hypertrophy, where the walls thicken axisymmetrically, and the lumen of the vessel narrows. Furthermore, prolonged high blood pressure can cause damage to the endothelial cells lining the inner surface of blood vessels. Endothelial dysfunction impairs the production of nitric oxide to prevent vascular relaxation and dilation. The loss of this vasodilatory effect contributes to increased vascular resistance and Young’s modulus.

Extensive evidence and data supporting these findings can be found in the relevant literature, and reported in Table 3. Similar to what reported above for the diabetic patient model, a calibration process was repeated for the hypertensive patient model to find better alignment with in vivo data from the literature. This led to the alignment of RI values with in vivo data at both early stage hypertension (in vivo 0.65 [8] vs. 0.66 openBF) and severe stage hypertension (in vivo 0.73 [11] vs. 0.71 openBF).

### Generation of Virtual Population

For the generation of virtual populations, firstly, parameters for each blood vessel were randomly sampled according to their physiological distribution as reported in Table 1, for each of the six age groups. Subsequently, we generated 12,000 virtual subjects, evenly distributed across the six age groups, with 2,000 subjects in each age group. To maintain physiological validity, we implemented a filtering process based on the methodology proposed by Benemerito et al. [69]. Specifically, we removed subjects whose mean values of systolic or diastolic brachial blood pressure deviated by more than 2.575 standard deviations from the experimentally measured mean values [38]. This filtering step allowed us to exclude parameterizations that would lead to unrealistic values, and introduce undesired bias into the results, to ultimately ensure alignment with physiological norms. The resulting number of virtual individuals for each age group is listed as *n* in Table 1. Because blood pressure ranges vary across age groups, the number of randomly selected physiologically appropriate age-based health individuals varies slightly within each age group. The filtering process resulted in a subset of 4167 physiological subjects from the initial 12,000. We then used the distribution of SBP, DBP, RI and mean renal blood volumetric flow rate at different ages to validate this ageing model.

To generate virtual populations representing patients with diabetes or hypertension, we scaled the parameters of each healthy subject within six different age groups by multiplying them by the normalised values for CO and vessel mechanical properties across all vessels. We assumed that the mechanical properties of all segments of the aorta followed the same trends reported in Tables 2 and 3 for specific aortic segments. This resulted in an equal number of virtual patients, for each disease type and stage, and for each of the six age groups to the number of healthy individuals within the same age group. To validate the disease model, we considered the range of 20-79 yo age group, for which in vivo data on the RI distribution for DN [70] and HN [8, 70] at early and severe stages were available.

## Results

### Validation of Ageing Model

**Fig. 3:**
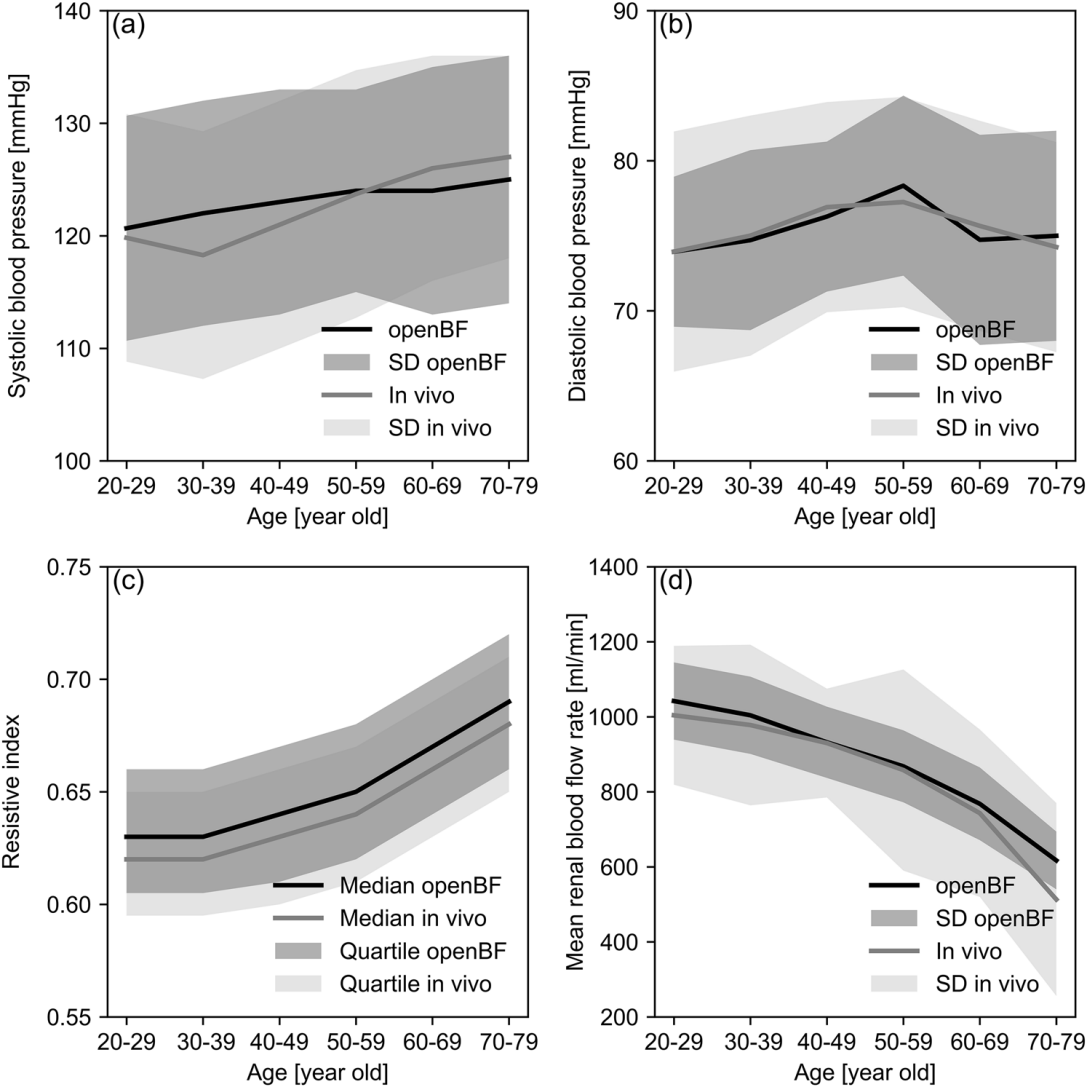
Validation of modelled data for a healthy, ageing population. **a** and **b**: Comparison of modelled systolic and diastolic blood pressure in the brachial artery against in vivo data [38]. **c**: Comparison of RI distributions in renal segmental arteries against ultrasound measurement data [71]. **d**: Comparison of modelled total mean renal blood flow rate (the sum of mean RBF rate in both kidneys) in main renal arteries against MRI-measured data [72]

Results reported in Fig. 3 show a comparison between predictions from openBF and in vivo data from literature. Figure 3a and b show good alignment of brachial systolic and diastolic blood pressure, for both mean trends and distributions and across the different age groups. For ages over 50 years old, diastolic pressure declines while systolic pressure continues to rise, and this is well captured by the modelled data. Figure 3c shows a comparison between model-derived RI data and in vivo data. Modelled and in vivo median RI values and age-specific distributions follow similar upward trends, ranging from 0.63 (0.62 for in vivo data) at 20–29 yo age group to 0.67 (0.66 for in vivo data) at 70–79 yo age group, with a small offset between modelled and in vivo data. Figure 3d shows a comparison between modelled and in vivo data for total mean RBF rate (the sum of mean RBF rate in both kidneys). OpenBF predictions show a reduction in blood volumetric flow rate, decreasing from approximately 1000 to 670 ml/min from ages 20 to 79. The results of openBF are generally in good agreement with in vivo data, although some differences are observed in the 20–29 yo age group (1003 ml/min for in vivo data against 1066 ml/min for openBF) and 70–79 yo age group (512 ml/min for in vivo data against 670 ml/min for openBF).

### Resistive Index in Healthy, Diabetic and Hypertensive Models

**Fig. 4:**
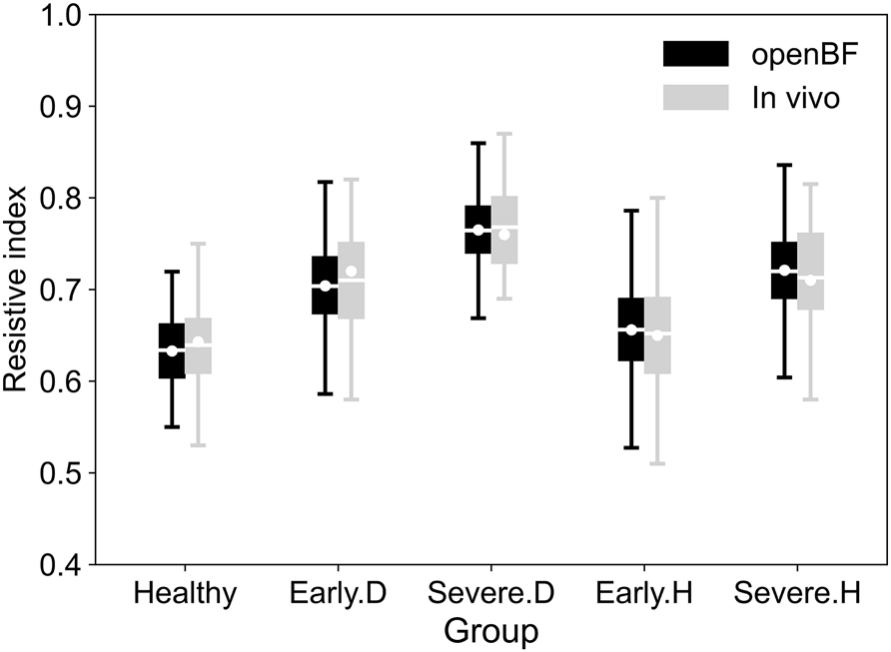
Comparison of RI values in segmental renal arteries with in vivo literature data for 20-79 yo healthy individuals [71], diabetic (Early.D/Severe.D) [70] and hypertensive (Early.H/Severe.H) patients [8, 70] at different disease stages. Simulation results are shown in black, and in vivo data are shown in grey. White solid lines represent mean values, while white solid circles represent median values

Figure 4 shows a comparison between predictions of RI from openBF and in vivo data from the literature for the 20–79 yo age group, in presence of healthy and disease. In general, this comparison shows good agreement for healthy, diabetic, and hypertensive populations and across the different stages of disease. The highest discrepancy (absolute percentage difference) between modelled and in vivo data in mean values was observed for groups representing healthy, early diabetic (Early.D), and severe hypertensive populations (Severe.H), where there were around 0.006 (1.02%) and 0.007 (0.99%), 0.008 (0.98%) difference between the openBF modelled and in vivo results, respectively. Some more noticeable difference between modelled and in vivo data was also observed in the minimum and maximum values for the healthy population, and severe hypertensive populations (Severe.H), where the discrepancy in maximum value among these groups were 0.030 (4.10%) and 0.022 (2.66%), respectively, and the discrepancy in minimum values among these groups were 0.030 (3.70%), and 0.020 (3.90%). Early.H have the highest discrepancy in the lower quartile with a discrepancy of 0.013 (2.10%), and Early.D have the highest discrepancy in the median value with a discrepancy of 0.022 (2.70%), and upper quartile with a discrepancy of 0.015 (2.00%). Furthermore, disease progression led to a significant increase in RI values for both diseases, with mean RI values increasing from 0.70 (Early.D) to 0.76 (Severe.D) for diabetes, and from 0.66 (Early.H) to 0.72 (Severe.H) for hypertension. Absolute percentage differences between openBF and in vivo results were computed as the difference between openBF values and in vivo values divided by their average, multiplied by 100.

### Biomarkers Discriminatory Performance in Healthy, Diabetic and Hypertensive Models

**Fig.5:**
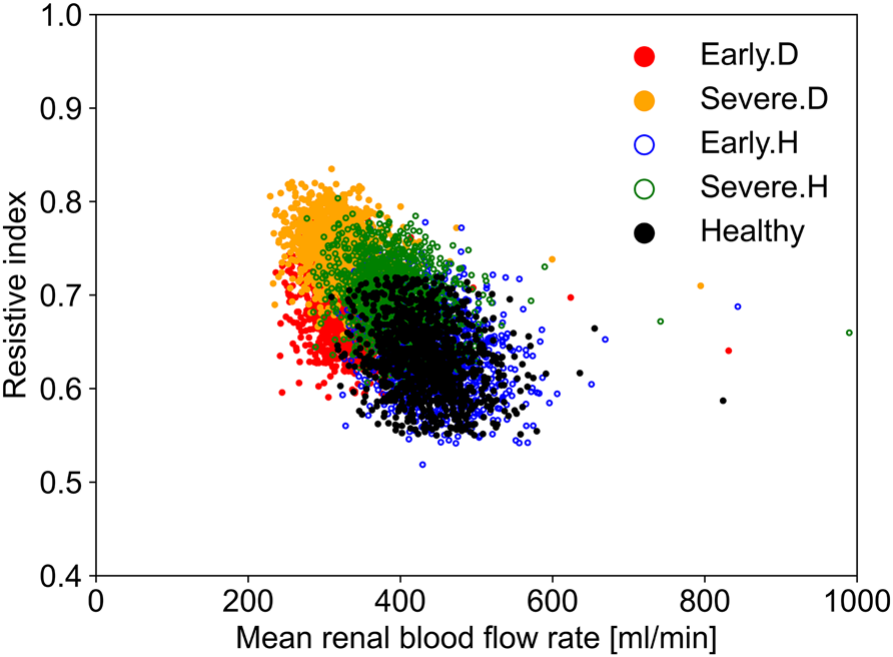
Scatter plot, showing RI and mean RBF rate distributions through single kidney for a 50-59 yo virtual population in presence of health, diabetes or hypertension at different disease stages

Figure 5 illustrates the distributions of RI and mean RBF rate through single kidney in the modelled healthy and diseased populations range between 50 and 59 yo. The distribution of RI from in vivo data indicate that RI might be a potential discriminator of disease progression, both for DN, where its values increased from 0.71 (SD 0.06) to 0.76 (SD 0.07) (as shown in Figure 4), as well as HN, with values increasing from 0.65 (SD 0.07) to 0.71 (SD 0.07), as they advance from the early to severe stages. However, the early and severe stages of diabetes and hypertension both show a substantial overlap. Distributions of RI and mean RBF rate in the modelled diseased populations for other age groups are provided as supplementary material, and show similar trends to those observed for the 50–59 yo age group.

**Fig.6:**
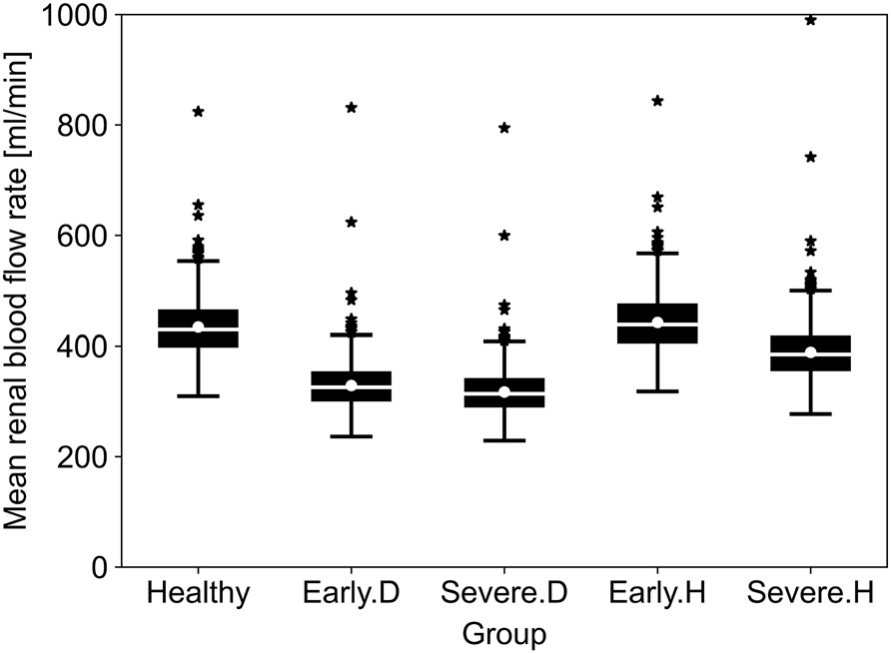
Box plot of modelled mean RBF rate through single kidney for 50 to 59 yo healthy individuals, DN and HN patients at different disease stages. White solid lines represent mean values, while white solid circles represent median values. Black solid star signs represent outliers defined as values that fall below the first quartile − 1.5 * interquartile range (IQR) or above the third quartile + 1.5 * IQR

Figure 5 illustrates that the mean RBF rate shows better disease stratification than RI between these two diseases at both early and severe stages. It is noticeable the significant overlap of the healthy population distribution with the distribution for the early stage of hypertension (Early.H). The distributions of mean RBF rate values through single kidney are also visualised via boxplots in Fig. 6, where its distribution with mean and standard deviation values for the Early.D and Early.H groups are 329 (SD 40) and 443 ml/min (SD 54), respectively. The interquartile range for Early.D is from 298 ml/min for the lower quartile and 354 ml/min for the upper quartile, mirroring the pattern observed in Severe.D. In Early.H, the interquartile range spans from 405 ml/min (lower quartile) to 476 ml/min (upper quartile), while in Severe.H, it ranges from 356 ml/min (lower quartile) to 420 ml/min (upper quartile). Notably, an overlap is evident around 380 ml/min between Early.D and Early.H. Similarly, an overlap is observed around 365 ml/min between Severe.D and Severe.H. This overlap results in a challenge to differentiate between DN and HN based solely on mean RBF rate.

### Receiver Operating Characteristic Curves

**Fig.7:**
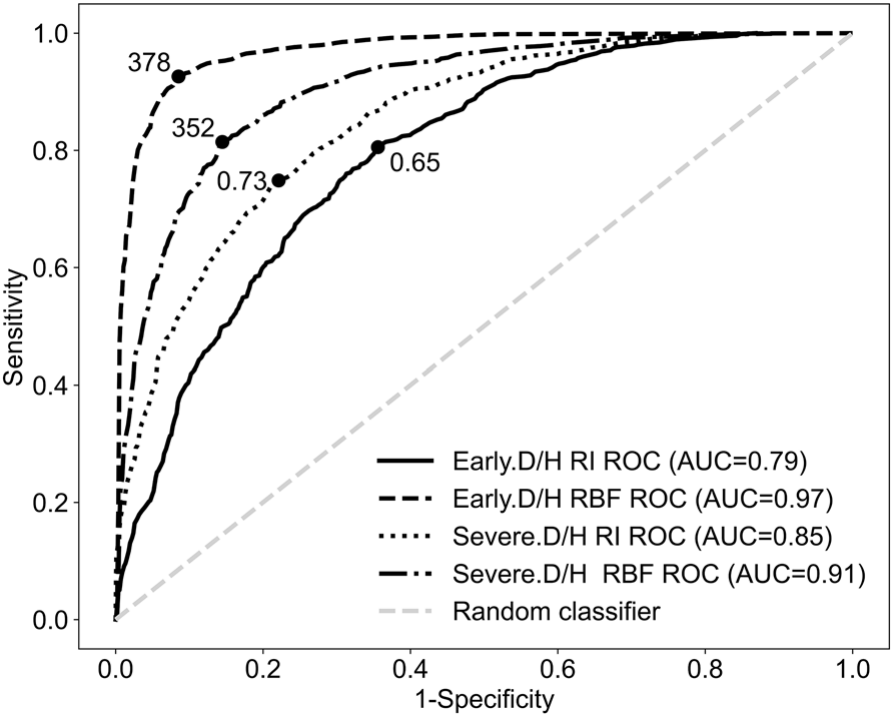
ROC curves for 50 to 59 yo Early.D, Early.H, and Severe.D, Severe.H populations using RI and mean RBF rate classifiers. Solid black line represents the ROC curve for the early disease stage (Early.D/H) using RI. Dotted black line represents the ROC curve for the severe disease stage (Severe.D/H) using RI. Dashed black line represents the ROC curve for the early disease stage (Early.D/H) using mean RBF rate. Dash dotted black line represents the ROC curve for the severe disease stage (Severe.D/H) using mean RBF rate. Black solid dot on each ROC curve is the best cut-off point to classify between DN and HN in early and severe stages. Dashed grey line represents a random classifier

Figure 7 presents the Receiver Operating Characteristic (ROC) curves for various classifiers, assessing and comparing the diagnostic performance of the biomarker RI or the mean RBF rate in distinguishing between DN and HN from early to severe stages. The ROC curve for the early disease stage (Early.D/H) using RI represents an area under the curve (AUC) of 0.79 (0.64 specificity, 0.81 sensitivity), indicating moderate diagnostic accuracy. In contrast, the ROC curve for the early disease stage (Early.D/H) using the mean RBF rate achieves a substantially higher AUC of 0.97 (0.91 specificity, 0.93 sensitivity), reflecting better diagnostic accuracy. For the severe disease stage (Severe.D/H), ROC curve using the RI represents an AUC of 0.85 (0.78 specificity, 0.75 sensitivity). Meanwhile, the ROC curve for the severe disease stage (Severe.D/H) using the mean RBF rate shows an AUC of 0.91 (0.86 specificity, 0.82 sensitivity). Additionally, Figure 7 marks the best cut-off values (solid black dots) for each ROC curve. The best cut-off point for the early disease stage is 0.65 for RI and 378 ml/min for mean RBF rate. Similarly, for the severe disease stage (Severe.D/H), the best cut-off point is 0.73 for RI and 352 ml/min for mean RBF rate.

In order to create ROC curves, all RI values and mean RBF rates in the early stages of kidney disease were considered potential cut-off points to distinguish patients with DN from those with HN. For each potential cut-off point, we calculated the true positive rate (sensitivity) and false positive rate (1-specificity). We repeated the same steps to generate the ROC curve for the severe stage. To determine the optimal cut-off point, we used Youden’s Index, which maximises the difference between sensitivity and specificity, calculated as the sum of sensitivity and specificity minus one. The cut-off point with the highest Youden’s Index was considered the optimal cut-off point for distinguishing between DN and HN patients.

## Discussion

The aim of this study was to develop a novel modelling approach, capable of realistically representing the effects of diabetes and hypertension in the kidney, and their progression, in typical arterial networks, and in presence of typical physiological variability observed in human populations. We have developed and extended an arterial network comprising a 1D representation of the biomechanics of blood flow through elastic vessels down to the interlobar arteries. The more peripheral renal arterioles were represented through lumped parameter models (arcuate, interlobular, and glomerular arterioles), which were fully coupled to the 1D whole-circulation model. A process of calibration was performed in order to find better alignment between modelled data and in vivo data in the presence of diabetes and hypertension.

The results show that this numerical approach can reliably model the ageing process of the cardiovascular system with strong quantitative alignment with in vivo results for DBP, SBP, mean RBF rate, and RI distribution in different age-based populations. For the validation of our ageing model, we used SBP and DBP data from the literature encompassing a total of 3,619 healthy men and women. As for the validation of our RI predictions, we used RI values from the literature reported for 572 healthy participants, aged 20 to 80 years. For validation of our mean RBF rate predictions, we used data from 180 healthy participants aged 20 to 90 years. The minor deviations observed between predicted and in vivo data from the literature can be attributed to variations in individual physiology, measurement methods, or limitations in the assumptions of the model. Furthermore, lifestyle choices, genetic factors, and underlying health conditions, not represented in our model, may also lead to differences between modelled and real data [73]. In contrast to existing models, our approach comprehensively accounts for the mutual influence of the renal system on the systemic circulatory system during the ageing process [23, 69]. This distinction is important, as the high-flow in the renal circulation and kidney can have significant influence on systemic blood pressure.

Model-derived and in vivo RI data (Fig. 3c) show a good agreement at population level. The age-specific trends, as evidenced by median values, exhibit similar trends to those observed in vivo, suggesting that openBF is capable of accurately capturing the effects of ageing and these pathologies on blood flow. The data showed a small offset between modelled and in vivo data for RI. This might be due to a simplistic representation of arterial biomechanics (Eq. 3) which does not consider viscous-elastic effects in the arterial wall. This may well affect a waveform-derived biomarker such as RI. Variations in the RI measurement locations for in vivo data, such as differences in renal artery branch selection or measurement techniques, may also lead to differences between in vivo and openBF results, where data was consistently extracted from specific locations, Fig. 2. The model also predicts a consistent reduction in mean RBF flow rate with ageing, similar to what is observed in vivo. However, a relatively high difference is observed in the 70–79 yo group, where openBF predicts higher flow rates when compared to in vivo data. This difference could be due to the fact that these in vivo data were reported for a group of individuals aged 80 and above, whereas in the openBF results, we only considered individuals aged 70 to 79 yo [72], for which parameterization data was available.

The simulations confirm that RI is a candidate biomarker to monitor the progression of kidney disease, with the mean value of RI increasing from 0.70 to 0.76 for early and severe stages of diabetes, and from 0.66 to 0.71 for early and severe stages of hypertension. For the validation of our Early.D and Severe.D populations, we used RI data from the literature encompassing one group of patients with diabetes and mild renal function impairment, and another group of patients with diabetes and moderate renal function impairment, reported for a total of 194 kidney disease cases, aged around 20 to 80. As for the validation of our Early.H and Severe.H populations, we used RI data from the literature reported for 132 hypertensive patients with mild renal function impairment, and a group of hypertensive patients with moderate renal function impairment, reported for a total 194 kidney disease cases, aged around 20 to 80. The model provides an explanation for these changes in RI by mechanistically linking these to direct disease effects. The increase in RI primarily results from narrowing of the vascular lumen and thickening of the arterial wall. The thickening of the arterial wall reduces the lumen area of the blood vessel, elevating vascular resistance and constraining the ability of blood vessel to dilate, thereby impacting diastolic blood flow stability. Consequently, to counteract this heightened resistance and sustain sufficient blood flow to the kidney, an increase in systolic blood velocity is necessary. Additionally, thickened arteries hinder passive expansion, reducing space for blood flow during diastole and causing decreased end-diastolic blood velocity. These factors lead to higher RI values by increasing peak systolic velocity and decreasing end-diastolic velocity.

The model also provides some mechanistic insight into the mean RBF rate changes with disease progression. In the early stages of diabetes, a decrease of 100 ml/min in mean RBF rate is observed in the Early.D population, as indicated by the parameterization of DN (refer to Table 2). A similar downward trend in total mean RBF rate is observed in stage 2, which is qualitatively in agreement with clinical data on kidney perfusion derived with arterial spin labelling (ASL) [15]. This decline is primarily attributed to decreased CO and increased renal peripheral vascular resistance resulting from diabetes. In the Early.H population, the distribution of mean RBF rate is similar to the healthy population. The model shows that this is due to the effect of two competing changes, the decreased lumen area of blood vessels leading to increased peripheral vascular resistance, and the increased CO. However, the model predicts a 60 ml/min decrease in the Severe.H population, characterised by a decrease in CO and continued reduction in lumen area alongside increased peripheral vascular resistance.

The scatter plot distribution (Fig. 5) reveals that there is a substantial overlap between healthy and disease distributions. However, compared to RI, mean RBF rate in diabetic and hypertensive populations exhibits better stratification performance for both early and severe stages. The mean RBF rate for individuals with early stage diabetes (Early.D) is concentrated within a lower interval (230 to 380 ml/min). In contrast, early stage hypertension (Early.H) data span a broader mean RBF rate (300 to 590 ml/min). Notably, this overlapping region enlarges as the disease progresses, indicating that as kidney disease worsens, distinguishing whether it is caused by diabetes or hypertension might be more challenging.

From the ROC curves in Fig. 7, it is evident that the AUC for Early.D/H using the mean RBF rate is significantly higher (0.97) compared to the AUC for Early.D/H using RI (0.79). This substantial difference in AUC indicates that the mean RBF rate has superior sensitivity and specificity in distinguishing early stage DN from HN. Similar trends are observed for RBF discriminating performance at severe stages of the two diseases. Although the difference in AUC is less pronounced in the severe stage, the mean RBF rate still demonstrates a higher accuracy and reliability. Therefore, the mean RBF rate shows promise as a more reliable biomarker for distinguishing DN from HN compared to RI, which is calculated based on systolic and diastolic velocities and serves as an indirect measure of renal vascular resistance. Further studies could look at integration of additional diagnostic criteria and biomarkers to improve the discriminating performance between disease types.

This study has some limitations to consider. Firstly, since the literature sources for model parameterisation tend to describe the effects of diabetic and hypertensive nephropathy only on large arteries such as the aorta and carotids, we had to assume that these pathologies affected the mechanical and geometrical properties the other vessels in a similar way. This may be true or there could be a differential effect between the more elastic versus the stiffer peripheral vessels. Furthermore, the study also does not consider the relatively common situation of subjects that have both diabetes and hypertension.

Secondly, our current study was based on a gender-neutral model. This approach utilised the parameterizations and distributions of vascular mechanical parameters in both sexes, ensuring that the results of the virtual individuals generated by our model were not biased toward either sex. However, this approach may result in an analysis of RI and mean RBF rate in virtual patients that lack sex-specific insight. Given that sex is a significant factor in clinical trials, it is recommended that in future studies a mechanistic model that incorporates sex-specific parameters is used. Additionally, the study did not account for variations in anthropometric features like height and weight.

Thirdly, the number of unphysiological virtual individuals filtered out in this study reached 7747 out of a total of 12,000 (65% filtering rate). The high rate of unphysiological virtual individuals in our model is primarily due to the random selection and allocations of parameters to our models. Although each parameter remains within physiological ranges, the independent parameters’ selection and their uncorrelated combination in a virtual patient can result in unrealistic combinations. For instance, a very stiff blood vessel wall might be paired with the same vessel being very narrow and long, therefore leading to unphysiological/unrealistic results (blood pressure). This issue is exacerbated by the complexity of the model, which involves over 100 blood vessels and 500 parameters. The unknown correlations between parameters across blood vessels significantly contribute to the low filter rate, as many generated combinations do not represent realistic physiological conditions. In the absence of more specific data distributions in real populations, we followed similar state-of-the-art approaches to Willemet et al. [74] and Benemerito et al. [69] for removing unrealistic parameterizations, which achieved similar filtration rates (58% and 75%, respectively).

In this study, the most peripheral renal vessels (arcuate, interlobular renal arterioles, and glomeruli) were not directly and individually modelled, but rather lumped into an R-C-R model representation. This prevented us from modelling the early differential effects of DN and HN, which mainly affect the glomeruli. When most patients first develop diabetic kidney disease, an increase in eGFR (over 120 mL/min/1.73m^2^) can be observed caused by dilation of afferent arterioles and constriction of efferent arterioles to response to diabetic kidney disease. This process is commonly and clinically known as hyperfiltration [75]. In hypertension, both afferent and efferent arterioles are constricted in the early stages [76]. These processes were not directly modelled in our study. In addition, the literature data that informed the parameterisation of our models was not detailed enough to separate the influence of solely diabetes or solely hypertension on the parameters of our model. These are often concomitant factors in chronic kidney disease. For example, the increased Young’s modulus observed in diabetic patients in the later stages of disease progression, might be caused by an increase in systemic pressure rather than by diabetes. Therefore, whether the increased Young’s modulus should be included as part of the parameterization of diabetic model remains worth investigating in the future.

In conclusion, we showed that our coupled 1D-0D computational model is capable of realistically capturing the physiological changes associated with ageing in healthy individuals and the renal damage resulting from diabetes or hypertension. By incorporating virtual patients representing various stages of diabetes and hypertension, our analysis indicates that mean RBF rate might help to differentiate between DN and HN from an early stage, whilst RI showed potential to be used in the progression of diabetic and hypertensive kidney disease. Following further validation and calibration in vivo, this modelling approach in renal circulation has the potential to identify biomarkers for clinical trials. These biomarkers could pave the way for a potentially non-invasive diagnosis and management of CKD from an early stage, when intervention may still be effective in mitigating or reversing the condition.
